# The Impact of Subject Age, Gender, and Arch Length on Attitudes of Syrian Dentists towards Shortened Dental Arches

**DOI:** 10.1155/2015/643176

**Published:** 2015-07-21

**Authors:** Mohammad Zakaria Nassani, Tammam Ibrahim Al-Nahhal, Omar Kujan, Bassel Tarakji, Elizabeth Jane Kay

**Affiliations:** ^1^Restorative Dental Sciences Department, Al-Farabi Colleges, P.O. Box 85184, Riyadh 11691, Saudi Arabia; ^2^Department of Removable Prosthodontics, Faculty of Dentistry, University of Aleppo, Aleppo, Syria; ^3^Oral and Maxillofacial Sciences Department, Al-Farabi Colleges, Riyadh, Saudi Arabia; ^4^Peninsula School of Dentistry, Plymouth University, Plymouth, UK

## Abstract

*Objective*. This study aimed to investigate the impact of subject age, gender, and arch length on dentists' attitudes towards unrestored shortened dental arches. *Materials and Methods*. 93 Syrian dentists were interviewed and presented with 24 scenarios for male and female subjects of different ages and shortened dental arches of varying length. Participants were asked to indicate on a standardized visual analogue scale how they would value the health of the mouth if the posterior space was left unrestored. *Results*. A value of 0.0 represented the worst possible health state for a mouth and 1.0 represented the best. The highest mean value (0.73) was assigned to a shortened dental arch with missing second molar teeth in the mouth of a 70-year-old subject. A 35-year-old female subject with an extremely shortened dental arch (all molar and premolar teeth are missing) attracted the lowest mean value (0.26). The statistical analysis indicated a significant decrease in the value placed on unrestored shortened dental arches as the number of remaining teeth decreased (*p* < 0.008). While subject gender had almost no impact on dentists' attitudes towards shortened dental arches, the scenarios for the older shortened dental arch subjects attracted significantly higher values compared to the scenarios for the younger subjects (*p* < 0.017). *Conclusion*. Subject age and arch length affect dentists' attitudes towards shortened dental arches, but subject gender does not.

## 1. Introduction

The shortened dental arch (SDA) concept aims at maintaining a functional dentition for middle-aged and elderly patients by concentrating treatment efforts at preservation and restoration of the strategically important parts of the dentition, that is, the anterior and premolar teeth, and avoiding extensive restorative treatment in the molar sites [1]. It has been argued that functional demands of modern man can be met despite the absence of molar support [2]. Furthermore, it has been reported that the prosthetic extension of the SDA by removable partial dentures could become a risk factor to the health of remaining oral structures [3].

The introduction of the SDA concept challenged the traditional concept of restorative dentistry in which prostheses and restorations are used to replace lost or seriously damaged parts of the dentitions. The SDA concept presents a cost-effective and simple approach in the management of partially edentulous dentitions [4]. Clinical studies have indicated that SDAs in which the anterior and premolar teeth are retained ensure adequate oral function in terms of chewing ability, aesthetics, stability of the dentition, temporomandibular joint function, and oral health-related quality of life [5–8]. In line with the SDA concept, the World Health Organization adopted as a goal for dental health “the retention throughout life of a functional, aesthetic, natural dentition of not less than 20 teeth and not requiring a prosthesis” [9]. However, the process of making a treatment decision for subjects with SDAs is not that simple. Many factors may interplay to affect the clinical decision whether to accept the mouth with a SDA as being healthy (as the WHO statement implies) or to extend the SDA with a prosthesis.

Käyser [1] indicated that barriers to undertaking extensive restorative treatment such as poor general health, poor oral health, poor motivation, or limited financial resources are decisive factors in favour of deciding not to extend the SDA with a prosthesis. On the other hand, extension of the SDA is positively recommended where loss of posterior teeth has created functional problems such as chewing impairment, occlusal instability, and/or aesthetic complaint [10, 11].

Among the factors that might be critical to treatment decision about SDAs are possibly subject age and gender. These factors were reported to have a significant impact on the way subjects perceive and evaluate their dentitions [12].

What remains unclear is the minimum number of teeth that is required to satisfy the long-term functional and social demands of subjects with reduced dentitions [13–15]. And yet fulfilment of patients' oral health ambitions and preferences is what drives dental attendance and dental treatment decision making.

Whilst many dental professionals in many countries accept that leaving an SDA unrestored with a prosthesis is an appropriate way to treat patients [16–22], it is acknowledged that common variables such as subject age, gender, and number of the remaining teeth would probably affect dentists' attitudes towards the SDA as a treatment option. However, the exact process of the evaluation underlying this decision path is unknown and hitherto unresearched.

One possible way to assess dentists' attitudes towards the different treatments is to measure the value they place on the outcome of treatment using utility measurements. According to Rohlin and Mileman [23] the concept of utility refers to the level of “desirability that people associate with a particular outcome.” The same authors indicated that “utilities are defined as numbers that represent the strength of a person's preference for particular outcome when faced with uncertainty.” To measure the utility of a health state, the best possible health outcome takes the value “1” whilst the worst possible health state is given the value “0.” Consequently, the values of any intermediate outcomes will fall between these two extremes of the utility scale. A number of utility methods have been developed and used in the medical and dental literature. These include the rating scale, the standard gamble, the time trade-off, and the willingness to pay [24, 25]. The relative merits, validity, reliability, and limitations of these methods have been reviewed by Froberg and Kane [26], whereas Matthews et al. [27] discussed their application in clinical decision making in dentistry and policy analysis. Strength of preference, or utility, is related to what a subject is prepared to sacrifice, but rating scales such as visual analogue scale (VAS) do not involve a “trade.” However, VAS scores/values have been shown to be well correlated with scales based on other methods (*r* = 0.56–0.65) [28].

The aim of this study was to investigate the impact of subject age, gender, and arch length on how dentists value the unrestored SDA as a treatment outcome in order to better understand the decision, evaluation, and outcome preferences of dentists when they make decisions and offer advice and treatment to subjects with SDAs.

The following hypotheses were examined:

 There are no significant differences in dentists' attitudes towards the unrestored SDAs among subjects of different ages.
 There are no significant differences in dentists' attitudes towards the unrestored SDAs among subjects of different gender.
 There are no significant differences in dentists' attitudes towards the unrestored SDAs of varying arch length.

## 2. Methods

The target population for this study was Syrian dentists studying postgraduate studies or working as part-time lecturers at the Faculty of Dentistry, The University of Aleppo.

A data collection sheet was designed and validated through a pilot study. The pilot study included 5 dentists. The aim of the study was explained and the dentists were requested to read and complete the data collection form. The dentists were encouraged to state their comments about the clarity of the described scenarios and the feasibility of the planned data collection form. Dentists' comments were noted and the data collection form was modified accordingly. The data collection sheet presented 4 SDAs of varying length, and each was presented as belonging to subjects of different genders and ages (male or female; 35, 50, or 70 years of age) (Table 1). The subjects were described as being healthy with no apparent problems with any of the remaining teeth or their supporting structures and with no signs or symptoms of temporomandibular joint dysfunction.

In order to measure the utility value placed on each SDA scenario, dentists were instructed to indicate on a standardized VAS how they would value the health of the mouth if the posterior space was left unrestored. The VAS was a 10 cm horizontal line with two clear end-points. The left end-point represented the worst health state or number zero. This point was labeled by the statement “the subject's mouth could not be worse.” On the other end of the line, the right-hand anchor or end-point represented the perfect health state. It was labeled by the statement “the subject's mouth could not be better.”

Dentists were asked to make a vertical mark on the line and at the point between the extremes which they felt represented the value of the scenario under consideration, in the subject described (male/female; 35, 50, or 70 years of age). The distance of the mark from the left-hand side of the VAS, in centimeters, divided by ten, comprised the utility value. The division by ten was in order that full health is represented by unity (value = 1) which is the accepted convention in utility measurement [28].

The collected data were analysed using the SPSS statistical package (IBM SPSS Statistics for Windows, Version 20.0, Released 2011, IBM Corp, Armonk, NY, USA).

The utility values for each SDA scenario were obtained by calculating a simple mean for the participant group of dentists as a whole. Paired samples *t*-tests were used to examine whether there were differences in dentists' utility values among the different SDA scenarios. Bonferroni's correction was applied to account for multiple comparisons and to set the significance level for each statistical comparison.

## 3. Results

Over the period of this investigation, 110 Syrian dentists were invited to participate. 93 dentists approved to complete the study form. 17 dentists declined participation; therefore, the final response rate was 85%.

The majority of participant dentists graduated from a Syrian university (93.5%). Their graduation year ranged between 1984 and 2011 (mean graduation year = 2005). 59% were male dentists and 41% were female. The mean age of participants was 29 years with a range between 22 and 50 years of age.

Dentists' mean utility values ranged between 0.26 for the 35-year-old female subject with an extremely SDA, where all molar and premolar teeth were missing, and 0.73 for the 70-year-old SDA subject with missing second molar teeth (Table 2).

In order to test the hypothesis of no significant differences in dentists' attitudes towards SDAs of varying arch length, the *t*-test for paired observations was used. The statistical analysis indicated a significant decrease in the value placed on unrestored SDAs as the number of remaining teeth decreased (*p* < 0.008) (Table 3). The hypothesis that arch length did not affect how dentists valued SDAs was therefore rejected.

The *t*-test for paired observations was also used to test the hypothesis of no significant differences in dentists' attitudes towards the unrestored SDAs among subjects of different ages. Tables 2 and 4 show that the scenarios for the older SDA subjects attracted significantly higher utility values compared to the scenarios for the younger subjects (*p* < 0.017). The hypothesis that subject age did not affect how dentists valued SDAs was therefore rejected.

Table 5 indicates that subject gender had almost no impact on the value placed on SDAs (*p* > 0.017). The hypothesis that subject gender would not influence how dentists valued SDAs was therefore accepted.

## 4. Discussion

Attainment of appropriate treatment decisions and efficient allocation of health budgets require thorough understanding, not only of treatment preferences of patients but also of those of the health care providers. This study was planned in order to gain more insight into dentists' evaluation of the unrestored SDAs under different clinical scenarios.

The sample of this investigation was a convenient sample. It comprised Syrian dentists working or undertaking postgraduate training in one of the Syrian dental schools. This is a limitation for this study as the surveyed sample was not intended to be a representative of either Syrian dentists in general or dentists in general. This flaw is accepted but we do not believe that it undermines the general concepts which the research has revealed.

To date, only a few authors have utilized utility measurement in dental research [12, 29–40]. Among the different utility methods reported in the literature, the VAS was chosen in this investigation to elicit dentists' utilities of the SDAs. This type of rating scale has been shown to have good intrarater and interrater reliability (*r* = 0.70–0.94 and *r* = 0.75–0.77, resp.). Test-retest reliability is also greater for rating scales than for other methods of utility measurement, such as standard gambles or time trade-offs [26, 34]. For this reason and because the rating scale is simpler and easier to understand by most people compared to other utility scales [41], the rating scale was chosen as a utility measurement method in this investigation.

Previous studies among dental professionals sought to assess merely their attitudes towards the SDA concept and its application in clinical practice [16–22]. The current study attempted to evaluate dentists' attitudes towards unrestored SDAs of varying length for male and female subjects of different ages. The impact of such factors on clinical decision making for subjects with SDAs has not previously been systematically evaluated in this way.

The results showed that dentists place lower values on unrestored SDAs as they get shorter. This accords with a recently published study that reported a decrease in dentists' evaluation of the SDAs as the number of posterior teeth lost increased [40].

From a subjective perspective, it has been reported that older subjects tend to assign higher utility values to the SDA state [12, 36]. In the current study, the impact of subject age on dentists' attitudes towards the unrestored SDAs was noticeable. The older the subject, the greater the value the dentist placed on the unrestored SDAs. It therefore seems that dentists are comfortable with the SDA concept in terms of their assessment of the functional needs of older subjects. The surveyed dentists therefore seem to believe that, in terms of treatment for elderly subjects, “less is more” and that a minimal intervention strategy is optimal. This may be because dentists believe that elderly subjects will have greater difficulty to attend long dental sessions or to undergo extensive dental restorative procedures compared to younger subjects. Alternatively, perhaps they consider that the oral environment, manual dexterity, and neuromuscular control may deteriorate with aging to a level which would render an elderly subject unable to manage removable dentures or to maintain the level of oral hygiene which fixed prostheses require [42, 43].

In a sample of British subjects, female subjects assigned much lower utility values to the unrestored SDA than male subjects [36]. However, no gender difference in the utility of the SDA was reported in a sample of Syrian subjects [12]. The results of our study show that subject gender has no impact on dentists' attitudes towards the unrestored SDAs.

In planning oral health policies, the values of both dental professionals and patients should be considered [30]. While the focus of this study was on general dental practitioners, it comes as a part of the ongoing research by the authors on the value of the SDAs among patients and dental professionals [12, 36, 37, 40].

It has been increasingly recognized that objective clinical measures of disease are insufficient as indicators of functional, psychological, and aesthetic aspects of oral health and, therefore, inadequate as indicators of treatment need. Rather, optimal treatment decision making, which leads to good patient care and enhanced patient satisfaction, is likely to be based on first class communication between dentist and patient [44]. It is therefore essential that studies such as the one presented here continue to explore the values that professionals (and patients) place on treatment outcomes.

The results presented offer some cause for concern as much as they indicate that an elderly subject who placed great value on having his/her mouth and SDA restored might find it difficult to persuade a dentist that this was in his/her best interest. It could therefore be argued that these data indicate a systematic discrimination against, and professional assumption of low level of need amongst, the most elderly sector of society. This should be of great concern as the first generation to have retained at least some of their dentitions is just becoming elderly/very elderly. This generation is one with high expectation of oral health, oral health services, and restorative techniques. If the results reported were translated into treatment decision making there are some issues which are worthy of consideration. The current generation of elderly subjects is already of concern because of their increasingly great numbers and the new challenges they pose to the dental profession in terms of the maintenance of compromised but highly valued dentitions. The results of this study may suggest that the dental profession may condemn the elderly population to treatment which would be suboptimal in the eyes of an old subject who has high expectations. Any type of ageist discrimination like this should be challenged, as all treatment decision should be made according to the merits and needs and preferences of the individual case, regardless of age. However, in younger patients posterior support is necessary for the long term stability of the occlusion, and in later years occlusal forces diminish, posterior teeth become less visible, and the functional demands on the dentition tend to decrease. These important clinical considerations may explain the results and suggest that dentists carefully weigh their patients' treatment requirements on the basis of their individual physiological and psychosocial needs.

By consideration of the impact of cross-cultural variations and professional characteristics on making treatment decisions [45, 46], future research among other cohorts of dentists from different cultural and professional backgrounds is recommended to confirm the findings of this investigation.

## 5. Conclusion

The results of this study indicate that the hypothesis that dentists values and attitudes towards unrestored SDAs are unaffected by subject age, can be rejected, but the hypothesis that subject gender has no effect on dentists attitudes to unrestored SDAs can be accepted. Finally, it is clear that arch length affects the value placed on SDAs by dentists.

## Figures and Tables

**Table 1:** Shortened dental arch scenarios used in this study.

SDA scenario	Length of the SDA	Label
Scenario 1	SDA with missing second molar teeth (upper and lower)	6 to 6

Subscenarios	Male SDA subject aged 35 years	
	Female SDA subject aged 35 years	
	Male SDA subject aged 50 years	
	Female SDA subject aged 50 years	
	Male SDA subject aged 70 years	
	Female SDA subject aged 70 years	

Scenario 2	SDA with missing molar teeth (upper and lower)	5 to 5

Subscenarios	Male SDA subject aged 35 years	
	Female SDA subject aged 35 years	
	Male SDA subject aged 50 years	
	Female SDA subject aged 50 years	
	Male SDA subject aged 70 years	
	Female SDA subject aged 70 years	

Scenario 3	SDA with missing molars and second premolars (upper and lower)	4 to 4

Subscenarios	Male SDA subject aged 35 years	
	Female SDA subject aged 35 years	
	Male SDA subject aged 50 years	
	Female SDA subject aged 50 years	
	Male SDA subject aged 70 years	
	Female SDA subject aged 70 years	

Scenario 4	SDA with missing molars and premolars (upper and lower)	3 to 3

Subscenarios	Male SDA subject aged 35 years	
	Female SDA subject aged 35 years	
	Male SDA subject aged 50 years	
	Female SDA subject aged 50 years	
	Male SDA subject aged 70 years	
	Female SDA subject aged 70 years	

SDA: shortened dental arch.

6: first molar; 5: second premolar.

4: first premolar; 3: canine.

**Table 2:** Dentists' mean utility values of the shortened dental arch scenarios.

Subject age	Gender	Length of the SDA			
		6 to 6	5 to 5	4 to 4	3 to 3
		Mean (SD)	Mean (SD)	Mean (SD)	Mean (SD)
35 years of age	M	0.65 (0.27)	0.49 (0.29)	0.40 (0.31)	0.28 (0.26)
	F	0.65 (0.28)	0.49 (0.31)	0.38 (0.32)	0.26 (0.36)

50 years of age	M	0.69 (0.28)	0.54 (0.26)	0.44 (0.27)	0.32 (0.32)
	F	0.71 (0.27)	0.53 (0.28)	0.43 (0.29)	0.30 (0.32)

70 years of age	M	0.73 (0.36)	0.61 (0.30)	0.51 (0.26)	0.38 (0.28)
	F	0.73 (0.35)	0.61 (0.30)	0.51 (0.27)	0.37 (0.29)

Utility values are within the range 0-1, where 0.0 = total lack of oral health and 1.0 = total oral health.

M: male.

F: female.

SDA: shortened dental arch.

SD: standard deviation.

6: first molar; 5: second premolar.

4: first premolar; 3: canine.

**Table 3:** Mean difference and standard deviation of the mean difference for paired *t*-test to examine the impact of arch length on dentists' attitudes towards SDAs.

Length of the SDA	Subject age					
	35 years of age		50 years of age		70 years of age	
	M	F	M	F	M	F
(6 to 6) versus (5 to 5)	0.16 (0.25)^*∗*^	0.17 (0.27)^*∗*^	0.14 (0.32)^*∗*^	0.17 (0.31)^*∗*^	0.12 (0.30)^*∗*^	0.12 (0.30)^*∗*^
(6 to 6) versus (4 to 4)	0.25 (0.35)^*∗*^	0.27 (0.35)^*∗*^	0.25 (0.41)^*∗*^	0.27 (0.43)^*∗*^	0.21 (0.44)^*∗*^	0.22 (0.45)^*∗*^
(6 to 6) versus (3 to 3)	0.37 (0.45)^*∗*^	0.39 (0.41)^*∗*^	0.37 (0.51)^*∗*^	0.41 (0.48)^*∗*^	0.34 (0.58)^*∗*^	0.36 (0.56)^*∗*^
(5 to 5) versus (4 to 4)	0.09 (0.17)^*∗*^	0.11 (0.16)^*∗*^	0.11 (0.19)^*∗*^	0.10 (0.24)^*∗*^	0.09 (0.26)^*∗*^	0.10 (0.28)^*∗*^
(5 to 5) versus (3 to 3)	0.21 (0.29)^*∗*^	0.22 (0.26)^*∗*^	0.22 (0.32)^*∗*^	0.23 (0.31)^*∗*^	0.22 (0.41)^*∗*^	0.24 (0.41)^*∗*^
(4 to 4) versus (3 to 3)	0.12 (0.19)^*∗*^	0.11 (0.16)^*∗*^	0.12 (0.22)^*∗*^	0.13 (0.17)^*∗*^	0.13 (0.26)^*∗*^	0.14 (0.26)^*∗*^

^*∗*^Significant difference at *p* < 0.05/6 = 0.008 (Bonferroni's correction).

M: male.

F: female.

SDA: shortened dental arch.

6: first molar; 5: second premolar.

4: first premolar; 3: canine.

**Table 4:** Mean difference and standard deviation of the mean difference for paired *t*-test to examine the impact of subject age on dentists' attitudes towards SDAs.

Subject age	Length of the SDA							
	6 to 6		5 to 5		4 to 4		3 to 3	
	M	F	M	F	M	F	M	F
35 versus 50	−0.04 (0.24)	−0.05 (0.23)	−0.05 (0.16)^*∗∗*^	−0.05 (0.18)^*∗∗*^	−0.04 (0.15)	−0.05 (0.14)^*∗∗*^	−0.04 (0.14)^*∗∗*^	−0.03 (0.13)^*∗∗*^
35 versus70	−0.07 (0.41)	−0.08 (0.43)	−0.11 (0.36)^*∗∗*^	−0.13 (0.39)^*∗∗*^	−0.11 (0.31)^*∗∗*^	−0.13 (0.32)^*∗∗*^	−0.11 (0.24)^*∗∗*^	−0.10 (0.24)^*∗∗*^
50 versus70	−0.04 (0.24)	−0.04 (0.28)	−0.07 (0.25)^*∗∗*^	−0.08 (0.27)^*∗∗*^	−0.08 (0.23)^*∗∗*^	−0.08 (0.24)^*∗∗*^	−0.07 (0.15)^*∗∗*^	−0.07 (0.16)^*∗∗*^

^*∗∗*^Significant difference at *p* < 0.05/3 = 0.017 (Bonferroni's correction).

M: male.

F: female.

SDA: shortened dental arch.

6: first molar; 5: second premolar.

4: first premolar; 3: canine.

**Table 5:** Mean difference and standard deviation of the mean difference for paired *t*-test to examine the impact of subject gender on dentists' attitudes towards SDAs.

Subject age	Males versus Female			
	Length of the SDA			
	6 to 6	5 to 5	4 to 4	3 to 3
35 years of age	−0.002 (0.11)	0.008 (0.10)	0.02 (0.07)^*∗∗*^	0.01 (0.05)^*∗∗*^
50 years of age	−0.01 (0.13)	0.008 (0.11)	0.004 (0.15)	0.02 (0.08)
70 years of age	−0.006 (0.05)	−0.006 (0.06)	0.003 (0.08)	0.01 (0.05)^*∗∗*^

^*∗∗*^Significant difference at *p* < 0.05/3 = 0.017 (Bonferroni's correction).

SDA: shortened dental arch.

6: first molar, 5: second premolar.

4: first premolar, 3: canine.
